# Changes in salivary biomarkers of burning mouth syndrome patients after clonazepam treatment

**DOI:** 10.22514/jofph.2024.019

**Published:** 2024-06-12

**Authors:** Sungil Jang, Ji-Eun Kim, Young-Hee Lee, Won Jung

**Affiliations:** Department of Oral Biochemistry, School of Dentistry, Institute of Oral Bioscience, Jeonbuk National University, 54907 Jeonju-si, Republic of Korea; Research Institute of Clinical Medicine of Jeonbuk National University—Biomedical Research Institute of Jeonbuk National University Hospital, 54907 Jeonju-si, Republic of Korea; Department of Clinical Laboratory Science, Wonkwang Health Science University, 54538 Iksan-si, Republic of Korea; Department of Oral Medicine, School of Dentistry, Institute of Oral Bioscience, Jeonbuk National University, 54907 Jeonju-si, Republic of Korea

**Keywords:** Burning mouth syndrome, Salivary biomarker, Salivary α-amylase, Clonazepam

## Abstract

There is a lack of objective indicators to evaluate the treatment effect of burning mouth syndrome, a neuropathic pain of unknown causes. Therefore, this study aimed to evaluate potential salivary biomarkers by analyzing saliva before and after clonazepam treatment in patients with burning mouth syndrome. Saliva was collected from 23 patients with burning mouth syndrome before and 4 weeks after the topical administration of clonazepam. Patients were classified as responders (pain relief of 50% or more, n = 10) or non-responders (n = 13) based on pain relief after treatment. Clinical examination data of responders and non-responders were compared using Mann-Whitney U test and Fisher’s exact test. Changes in the level of salivary biomarkers (salivary α-amylase, cortisol, calmodulin, α-enolase and interleukin-18) were evaluated before and after treatment using Wilcoxon signed-rank sum test, and their association with treatment response was examined using Fisher’s exact test. The salivary biomarker levels showed no significant differences between the responders and non-responders. However, the change in salivary α-amylase activity after treatment revealed a significant difference between the two groups (*p* = 0.039). Although not all patients showed the same pattern, there was a difference in the alteration of salivary α-amylase activity before and after treatment between responders and non-responders. Further study is required to clarify whether there is a causal relationship between salivary α-amylase activity and treatment response. However, considering that salivary α-amylase activity is related to orofacial pain and psychological stress, this suggests the potential use of salivary α-amylase as a biomarker for burning mouth syndrome.

## 1. Introduction

Chronic orofacial pain can affect the quality of life [1]. Burning mouth syndrome (BMS) is a type of chronic orofacial pain. BMS is a chronic disease that presents with a burning sensation without evidence of pathological changes in the oral mucosa [2, 3]. Although the cause of the disease is unknown, the international classification of orofacial pain classifies it as chronic neuropathic pain [4, 5].

BMS predominantly affects pre- and postmenopausal women and can affect anywhere in the oral mucosa; however, the most common sites are the anterior two-thirds of the tongue [3, 4]. The burning sensation can also be expressed as tingling, prickling or numbness. The intensity of pain varies from mild to moderate, but it usually does not interfere with sleep [3, 6].

Diagnosis and treatment of BMS are difficult because there are no pathological changes in the painful mucosa. Moreover, the lack of understanding of its pathogenesis makes it more difficult [2, 7, 8]. Consequently, diagnosis and treatment are being conducted based on the clinician’s experience. Although several studies have reported the effectiveness of various BMS treatment strategies, there is no effective treatment protocol for complete pain remission [8, 9, 10]. Among the various treatment strategies, clonazepam has been used as a first-line treatment for BMS [8, 11]. Because of these difficulties, BMS symptoms are often exacerbated by misdiagnosis and inappropriate treatment selection [7]. Therefore, research on monitoring the severity of diseases according to the course of treatment is necessary [12].

Salivary diagnostic methods enable early diagnosis and are useful for post-treatment monitoring [13]. Saliva is present in the affected area of BMS, and it has the advantage of being collected non-invasively and examiner-independent [13, 14]. Several salivary biomarkers (salivary α-amylase (sAA), cortisol, calmodulin, α-enolase and interleukin (IL)-18) which respond to inflammation, peripheral nerve damage, and stress are known to be elevated in BMS patients [15, 16, 17, 18, 19].

Therefore, this study aimed to investigate the correlation between salivary biomarkers and treatment outcomes by measuring biological markers in the saliva of patients with BMS before and after treatment. In addition, we aimed to evaluate the potential of these biomarkers as therapeutic diagnostic indicators for objective assessment of BMS in the future.

## 2. Materials and methods

### 2.1 Subject

BMS was clinically diagnosed according to the International Classification of Orofacial Pain diagnostic criteria [4]. Patients with oral burning pain without identifiable pathological lesions and with normal unstimulated salivary secretion were selected. Normal salivary secretion criteria included an unstimulated whole saliva flow rate of 0.1 mL/min or more [20]. The inclusion criteria included burning pain: occurring every day, recurring for 2 or more hours per day, and tongue affected.

Patients with burning pain attributable to identifiable causes were excluded. The exclusion criteria were as follows: history of head and neck malignancy, history of chemotherapy and/or radiotherapy, Sjögren’s syndrome, contact allergies, angiotensin-converting enzyme inhibitor use, thyroid disease, herpes zoster. Patients receiving pharmacological treatment for sleep disorders were also excluded. Patients with pathological visual changes in the oral mucosa such as redness, atrophy, ulcers, oral lichen planus, oral candidiasis, were also excluded. In addition, patients who have received BMS treatments in the past were also excluded.

All patients with BMS provided baseline saliva samples and information on various clinical variables. They were treated with topical clonazepam (0.5 mg) twice daily for 4 weeks. Post-treatment saliva samples were collected again. After treatment, patients were classified into two groups according to the extent of pain relief reported: responders (pain relief of 50% or more) and non-responders (pain relief less than 50%). We analyzed the saliva samples from the two groups to assess changes in salivary biomarkers and compared the clinical variables between the groups. A flowchart of the study is shown in Fig. 1.

**Fig. 1.** Study-flow diagram. (a) An overall schematic of the study design. Baseline saliva samples and clinical data were collected from BMS patients before topical application of clonazepam for 4 weeks. After treatment, patients were classified as responders or non-responders based on the extent of patient-reported improvement, and post-treatment saliva was collected. (b) The collected saliva was analyzed for changes in biomarkers before and after treatment. The association between the changes in salivary biomarker level and treatment responsiveness was analyzed.

The size of the study population was determined by power analysis using G*Power software version 3.1.9.7 (The G*Power Team, Düsseldorf, Germany) [21]. It was predicted that 12 or more participants would be required to detect a biomarker of which the salivary level is affected by treatment with an effect size of 0.8, 80% power, and alpha level at 0.05, using the Wilcoxon signed-rank sum test. In addition, it was predicted that 20 or more participants would be required to detect a correlation between patients’ response to treatment and change in salivary biomarker levels with an anticipated response rate of 50%, an expected proportion of increase and decrease of salivary biomarker level of 0.8:0.2 or *vice versa*, 80% power, and alpha level at 0.05, using Fisher’s exact test. Therefore, we planned to recruit at least 20 participants for the study.

### 2.2 Clinical data collection

Demographic characteristics and medical histories of the patients were recorded. The recorded characteristics of the burning pain included location, pattern, and severity. Pain severity was recorded using a numerical rating scale (NRS). Data on other symptoms accompanying the burning pain, including dysgeusia and dryness, were also collected. Sleep disturbance statuses were recorded based on self-reports from the patients describing whether they have sleep disturbance or not. If a patient answered that she had a significant stressors or events (regardless of physical or psychological) around the onset of the burning pain during the interview, then the patient was classified to have a stressor.

### 2.3 Saliva collection

Saliva samples were collected twice; before and after treatment. Saliva samples were collected using standardized protocols [15]. Saliva was collected between 9:00 AM and 12:00 PM. Patients were asked not to eat, smoke, or rinse their mouths from an hour before saliva sampling. The unstimulated whole saliva flow rate was measured while collecting saliva. The collected samples were immediately transported to a laboratory and processed. The samples were centrifuged at 2500×g for 20 min at 4 °C to separate the supernatant from the debris. The supernatant was stored at −80 °C until use. Samples were processed and stored within an hour of collection.

### 2.4 Salivary biomarker measurement

The salivary concentrations of sAA, cortisol, calmodulin, α-enolase, and IL-18 were measured using enzyme-linked immunosorbent assay (ELISA). Commercial ELISA kits were used to measure sAA (Abcam, Cambridge, UK, #ab137969), cortisol (Abnova, Taipei, Taiwan, #KA1885), calmodulin (Novus Biologicals, Centennial, CO, USA, #NBP2-74794), α-enolase (Abcam, Cambridge, UK, #ab181417) and IL-18 (R&D systems, Minneapolis, MN, USA, #DL180). Measurements and data analyses were performed according to the manufacturer’s instructions.

### 2.5 Statistical analysis

Statistical analyses were performed using SPSS software version 25 (SPSS Inc. Chicago, IL, USA). According to the characteristics of the variables, the Mann-Whitney U test or Fisher’s exact test was used to analyze the differences between the groups. The Wilcoxon signed-rank test was used to compare pain NRS scores and the levels of salivary biomarker candidates before and after treatment. Statistical significance was defined at *p *< 0.05.

## 3. Results

### 3.1 Patients and clinical examination

Saliva samples were collected from the 31 patients. Among the patients, eight did not attend the follow-up session and were excluded from this study. Finally, 23 pairs of saliva samples were collected from the 23 patients before and after treatment and used for further analyses. All patients were female, and their ages ranged from 52 to 82 years, with a median age of 65 years. All the patients complained of burning pain in the tongue. The other clinical findings are summarized in Table 1.

**Table 1.** Demographic and clinical examination data.

Variables		Responders (n = 10)	Non-responders (n = 13)	*p* value
Age (yr, median (range))		64 (55–78)	66 (52–82)	0.530^a^
Pain location				
	Tongue (%)	10 (100)	13 (100)	
	Lip (%)	2 (20)	4 (31)	0.594^b^
Accompanying symptoms				
	Dryness (%)	5 (50)	8 (62)	0.515^b^
	Dysgeusia (%)	1 (10)	5 (38)	0.119^b^
Unstimulated whole saliva flow rate (mL/min, median (range))		0.30 (0.20–1.00)	0.20 (0.15–0.80)	0.254^a^
Presence of stressor (%)		5 (50)	5 (38)	0.666^b^
Sleep disturbances (%)		5 (50)	4 (31)	0.666^b^
Pain increase by food (%)		6 (60)	6 (46)	0.231^b^
Pain pattern				
	Continued all day long (%)	6 (60)	8 (62)	0.889^b^
	Increase in the afternoon (%)	1 (10)	3 (23)	
	Decrease in the afternoon (%)	1 (10)	0 (0)	
	Night and during sleep (%)	1 (10)	1 (8)	
	Intermittent (%)	1 (10)	1 (8)	

*^a^, Mann-Whitney U test; ^b^, Fisher’s exact test.*

### 3.2 Treatment outcomes

Before treatment, the NRS scores reported by patients ranged from 2 to 8, with a median of 2. After 4 weeks of medication, all patients reported a similar or reduced NRS score (median: 1; range: 0 to 6; *p *< 0.001) (Fig. 2a). None of the patients reported an increased NRS score after treatment. Based on the extent of patient-reported improvement after treatment, 10 patients (43%) were classified as responders, and 13 patients (57%) were classified as non-responders. Patients in the responder group reported NRS scores of 2 or 3 (median: 2) before treatment, and the scores ranged from 0 to 2 (median: 1; *p* = 0.004) after treatment (Fig. 2b). The NRS scores reported by the non-responder group ranged from 2 to 8 (median: 3) before treatment, and the scores were distributed from 0 to 6 (median: 2) after treatment (*p* = 0.003; Fig. 2c).

To examine the effect of the administration of clonazepam on changes in salivary biomarkers, we measured the salivary concentration of sAA, cortisol, calmodulin, α-enolase, and IL-18 using ELISA. While sAA, cortisol, and calmodulin were measured in 23 pairs of saliva samples, α-enolase and IL-18 were measured in 22 pairs as we had run out of the samples after measuring the former 3 markers. After ELISA, samples with values that did not fall within the measurement range were excluded from further analyses. Descriptive statistics of each salivary biomarker is described in Table 2. No significant changes were observed in the salivary concentrations of the five markers after treatment (Fig. 3a–e). These results suggest that treatment with clonazepam, though it could improve patients’ pain sensation, did not affect the activity or concentration of these salivary biomarkers.

**Fig. 2.** Changes in patient’s pain after treatment. Changes in pain during treatment reported by (a) all patients, (b) responder group, and (c) non-responder group. Each patient was asked to report her pain based on NRS at the first visit and after treatment. Each dot indicates an NRS score reported from an individual patient at each visit. NRS scores at the first visit and after treatment were compared using Wilcoxon signed-rank sum test. NRS: numerical rating scale.

**Table 2.** Descriptive statistics of salivary biomarkers of BMS patients at the first visit.

Variables (unit)	First visit	After treatment
sAA (U/mL)	80 (1–658)	134 (8–551)
Cortisol (ng/mL)	2 (0–162)	2 (1–13)
Calmodulin (ng/mL)	0.093 (0.000–4.854)	0.040 (0.000–4.114)
α-enolase (U/mL)	154 (17–1116)	113 (56–2928)
IL-18 (pg/mL)	68 (43–833)	74 (53–449)

*All data are presented as mean and range. sAA: salivary α-amylase; IL-18: interleukin-18.*

**Fig. 3.** Effect of clonazepam treatment on salivary biomarkers. Concentration of (a) sAA, (b) cortisol, (c) calmodulin, (d) α-enolase, and (e) IL-18 was measured by ELISA, using saliva collected at the first visit and after treatment. Each dot indicates saliva collected from an individual patient. Upper and lower borders of a box indicate the third and the first quartile, with a thick line inside the box indicating a median. Upper whisker indicates 1.5 interquartile range, while lower whisker indicates the lowest observation. Concentrations of each biomarker at the first visit and after treatment were compared using Wilcoxon signed-rank sum test. sAA: salivary α-amylase; IL-18: interleukin-18.

### 3.3 Association between pain relief and change of salivary biomarker levels

Next, we assessed the relationship between changes in salivary marker levels and pain relief in both groups after medication. To address this, we classified changes in the levels of salivary markers as increased or decreased. Fisher’s exact tests were conducted to examine the association between the patient groups (responders and non-responders) and salivary markers. Among the five salivary markers measured, only the change in sAA activity was associated with pain relief; a decrease in sAA activity was associated with the responder group, whereas an increase in sAA activity was associated with the non-responder group (*p* = 0.039, Table 3). For the other salivary markers, no significant associations were found between changes in salivary biomarker levels and pain sensation (Table 3).

**Table 3.** Association between changes in salivary biomarker levels and changes in pain sensation.

Marker (n)^a^		Responders	Non-responders	*p* value^b^
sAA (23)				
	Increase	4	11	0.039
	Decrease	6	2	
Cortisol (23)				
	Increase	6	9	0.685
	Decrease	4	4	
Calmodulin (18)				
	Increase	2	2	0.569
	Decrease	4	10	
α-enolase (21)				
	Increase	5	4	0.670
	Decrease	5	7	
IL-18 (22)				
	Increase	4	4	1.000
	Decrease	6	8	

*^a^, Sample was excluded from analysis if it was run out or the measured value did not fall within the measurement range; ^b^, Fisher’s exact test. sAA: salivary α-amylase; IL-18: interleukin-18.*

## 4. Discussion

BMS is considered a neuropathic pain condition involving multiple potential etiologies and therefore may have multiple subtypes [2, 3, 6]. There are no diagnostic criteria or effective treatments for BMS due to a lack of understanding of its pathogenesis [2, 5]. Clonazepam is an effective medicine as the first-line treatment option, although there is no cure for the complete remission of BMS [8, 9, 11]. In this study, the subjective pain scores (NRS) of patients were significantly decreased after treatment with clonazepam, though not all patients showed a satisfactory response.

Therefore, the management of BMS is challenging for clinicians [3, 7]. Clinical variables, including salivary flow rate and pain location, may provide clues about the BMS subtype and could potentially influence treatment outcomes. Thus, we questioned whether there were differences in pain reduction after clonazepam treatment that were correlated with clinical variables. However, we could not find any significant differences in clinical variables between responders and non-responders. No significant difference in salivary flow rate was observed between responders and non-responders. This may be because patients with hyposalivation were excluded. Considering that hyposalivation is one causative factor for oral burning sensation [6, 20], further study on the association between salivary flow rate and treatment responsiveness may provide a valuable insight on the pathogenesis of BMS. Studies on the association between pain location and BMS pathogenesis have been rare. Given the high prevalence of tongue involvement as a primary site in BMS [4, 6], we set the tongue involvement as one of the inclusion criteria of this study. As a result, all participants reported pain of the tongue, with a minority had burning pain on the lips in addition to the tongue. The small sample size of this study and even smaller number of patients having pain on the site other than tongue might not be sufficient to examine the association between pain site and treatment responsiveness. Further studies with larger sample sizes are needed in the future.

Objective indicators that reflect the degree of BMS are required to evaluate treatment response in patients with BMS. Salivary biomarkers have several advantages as objective indicators of BMS. Saliva is typically in anatomical proximity to a place with a burning sensation. Moreover, the saliva collection method is easy, non-invasive, and requires no special skills [13, 14]. Previous studies have suggested that several salivary proteins, of which levels increased in BMS patients compared to controls, could act as biomarkers for BMS. However, to determine whether salivary biomarker candidates reflect the treatment response of patients with BMS, it is necessary to analyze salivary biomarkers before and after treatment. Therefore, we collected and analyzed the saliva of patients with BMS before and after treatment. Considering that no objective indicator exists, we attempted to identify a new biomarker that could reflect patients’ satisfaction with pain relief.

Unfortunately, there were no salivary biomarkers associated with treatment responses after treatment, except for sAA, in this study. The change in sAA activity after treatment was associated with the response to the treatment. sAA is one of the most abundant components of saliva. sAA, a digestive enzyme, is important for immunity because it prevents bacterial adhesion to the oral mucosa [22]. sAA is known to increase in patients with BMS [17, 19, 23]. Although sAA was increased in BMS patients, sAA has several reasons to act as a high-potential biomarker in BMS.

First, sAA is considered a highly sensitive biomarker of psychological stress, and BMS is believed to be closely related to stress [19, 24]. The concentration of sAA increases under physical and psychological stress, such as cold-water exposure and academic examination [25, 26, 27]. Stress, the sympathetic nervous system (SNS), and sAA are closely related. sAA is stress-dependent, and its secretion increases as the activity of SNS changes [28]. sAA was activated or increased in concentration under sympathetic responses [25, 26]. Several studies showed that the stimulus of direct β-adrenaline receptors regulated the synthesis and release of the sAA [24]. Stress causes an activation of the hypothalamic-pituitary-adrenal (HPA) axis and the SNS, which causes the secretion of sAA [29]. Several studies have reported an increase in the concentration and activation of sAA during stress [30, 31, 32]. Takai *et al*. [33] reported that sAA was significantly increased under watching a stressful video, whereas relaxation videos induced significantly decreased sAA. As such, sAA responds sensitively to the increase or decrease in stress. These results indicate that sAA is a valuable biomarker for stress-related diseases. The measurement of sAA can provide a method for evaluating the effectiveness of management interventions for stress-induced diseases [28].

Moreover, sustained pain, especially chronic pain with unknown causes, such as BMS, is associated with psychological distress [2, 6, 34]. Symptoms of BMS could have a major impact on the quality of life, and BMS is often accompanied by various mood disorders such as anxiety and depression [4, 35, 36]. Increased psychological distress is another factor that increases sAA. Psychosocial factors can affect perceived pain intensity and treatment response. In patients with chronic pain, excessive sAA levels may result from increased sAA activity owing to psychological distress [37].

Second, pain can induce the increased secretion of sAA [24]. BMS is a representative chronic neuropathic pain of the orofacial region [5, 38]. Generally, painful stimuli can be involved in sAA secretion by activating the sympathoadrenal medullary and HPA axes. In patients with chronic pain, sAA levels were positively correlated with pain intensity reported with visual analogue scale, and epidural block significantly reduced sAA level. While in pain-free group, there was no significant change in sAA levels after epidural block [37]. This result suggests that sAA can be a useful biomarker for evaluating pain intensity in patients with chronic pain. BMS is associated with psychosocial distress at disease onset. These characteristics suggest that sAA, which reflects the intensity of stress and pain, is a potential biomarker for prognosis and process assessment in BMS.

However, there were no significant differences between the groups in the remaining biomarkers except for sAA. IL-18 is a pro-inflammatory cytokine considered a potential factor in the immune response [39]. Ji *et al*. [15] reported that salivary IL-18 could be a potential biomarker in patients through proteomic analysis. Salivary cortisol levels have been used as a measure of HPA axis activity [40]. It was also reported to be increased in BMS patients, suggesting its potential as a biomarker [17]. Calmodulin is a component of the neutrophil signaling pathway [41]. Krief *et al*. [18] investigated the involvement of the neurotrophin signaling pathway in the pathophysiology of BMS by analyzing the salivary proteins of BMS patients. The detected signaling proteins were overexpressed in patients with BMS. In particular, calmodulin was only detected in patients with BMS. α-Enolase is a multifunctional surface protein that may play a role in the pathophysiological processes of autoimmune diseases [42]. α-Enolase was proposed as a potential biomarker for BMS by Ji *et al*. [15] as a result of proteomics in the saliva of patients with BMS. In the subsequent receiver operating characteristic (ROC) analysis based on ELISA validation data, α-enolase showed higher specificity and sensitivity. However, in the present study, none of these candidate biomarkers reflected the treatment progress of patients with BMS. This is thought to be because previous studies were case-control studies, whereas this study evaluated the progress of BMS according to treatment response.

This study had several limitations. We did not perform laboratory tests to rule out secondary BMS. Instead, we conducted careful oral examination to screen patients with oral mucosal abnormalities or possible secondary BMS, and excluded these patients from this study. Screening laboratory tests for all patients may have to be required in future studies to exclude secondary BMS more precisely. This study was also limited by the small number of patients, the lack of a control group, and the short follow-up period. In addition, regarding a patient with a dissatisfied response at the end of the treatment period as a non-responder has limitations because the response duration varies among patients, and there is no clear treatment protocol [2, 3, 8]. These limitations restrict the generalizability of the results of this study. Nevertheless, the results of this study, which analyzed saliva before and after treatment in patients with BMS, showed that sAA is a potential BMS biomarker for monitoring treatment response. Therefore, repeated measurements are required over a longer follow-up period with a larger number of participants to confirm that sAA is a useful biomarker for evaluating BMS progression.

In this study, change in sAA activity was found to be associated with the treatment response. It is still difficult to confirm it as a biomarker for BMS because it did not show a significant difference after treatment in all patients with BMS. However, considering that the only clinical evaluation method for a BMS is subjective pain, the use of sAA that reflects pain is expected to have more potential in the future. In particular, sAA has been reported to be associated with pain, psychosocial stress, and sympathetic dysregulation, suggesting its potential use as a biomarker for BMS.

## 5. Conclusions

• Salivary biomarkers examined in this study showed no significant differences between the responder group and the non-responder group.

• The change in sAA activity differed according to the treatment response.
